# Counting Rankings of Tree-Child Networks

**DOI:** 10.1007/s11538-026-01606-6

**Published:** 2026-02-21

**Authors:** Qiang Zhang, Mike Steel

**Affiliations:** Biomathematics Research Center, School of Mathematics and Statistics, University of Canterbury, Christchurch, New Zealand

**Keywords:** Phylogenetic network, Algorithm, Rankings, Enumeration

## Abstract

Rooted phylogenetic networks allow biologists to represent evolutionary relationships between present-day species by revealing ancestral speciation and hybridization events. A convenient and well-studied class of such networks are ‘tree-child networks’ and a ‘ranking’ of such a network is a temporal ordering of the ancestral speciation and hybridization events. In this short note, we investigate the question of counting such rankings on any given binary (or semi-binary) tree-child network. We also investigate the relationship between rankable tree-child networks and the class of ‘normal’ networks. Finally, we provide an explicit asymptotic expression for the expected number of rankings of a tree-child network chosen uniformly at random.

## Introduction

Rooted phylogenetic networks provide an effective model for biologists to represent the relationship between present-day species and their common ancestor through speciation and hybridization events Huson et al. (2010); Marcussen et al. (2014). Tree-child networks are a class of phylogenetic networks where each ancestral species has at least one path to the present via speciation events Cardona et al. (2008). For some tree-child networks, it is possible to impose a time-stamp on each species in such a way that (i) earlier species are assigned earlier time-stamps than their non-hybrid descendants, and (ii) hybrid species are assigned the same time stamp as their parents. This assignment of time-stamps gives rise to a discrete temporal ‘ranking’ of the vertices of the network. This leads to some natural questions, such as: ‘Does a given tree-child network *N* have a temporal ranking?’, ‘If so, how many different temporal rankings does *N* have?’, and ‘What is the average number of temporal rankings of a tree-child network chosen uniformly at random?’.

The answer to the first question can be no for certain tree-child networks Baroni et al. (2006), and in this paper, we further investigate the relationship between the existence of a ranking and the class of ‘normal’ networks. We then introduce a new method to address the second question (i.e., to count the number of temporal rankings of any given separated tree-child network). Finally, we address the third question by deriving an asymptotic expression for the expected number of rankings of a tree-child network chosen uniformly at random.

### Definitions and notation

A *simple directed acyclic graph* is a directed graph with no directed cycles, no self-loops, and no multiple arcs between the same ordered pair of vertices. Let $D=(V,A)$ be a simple directed acyclic graph. *D* is connected if there is a path (ignoring arc directions) between any two vertices. A *descendant* of a vertex *v* is any vertex *u* that can be reached by following a directed path (possibly reduced to a single vertex) from *v*, denoted as $v \preceq u$. We write $v \prec u$ if $v \preceq u$ and $v \ne u$. A *topological ordering* of *D* is an ordering of all vertices $\tau =(v_1,v_2,\cdots , v_n)$ with the property that if there is a directed path from $v_i$ to $v_j$, then $i<j$. Define the *index* induced by $\tau$ of a vertex *v* by $\text {ind}_{\tau }(v_i)=i$. Note that a topological ordering is a total ordering of all vertices (i.e., any two vertices are comparable). Let $\delta (D)$ denote the number of topological orderings of *D*, and since *D* is acyclic, we have $\delta (D)\ge 1$ (e.g., by Proposition 1.4.3 of Bang-Jensen and Gutin (2001)).

A *rooted network* is a connected simple directed acyclic graph $N=(V,A)$ such that each vertex is either

- a *root vertex* of in-degree 0 and out-degree at least 2;
- a vertex of in-degree 1 and out-degree at least 2;
- a *reticulate vertex* of in-degree $> 1$;
- a *leaf* of in-degree 1 and out-degree 0.

A *tree vertex* is a vertex that is not a reticulate vertex. A *tree-to-tree vertex* is a tree vertex that has tree vertices as children. A leaf is not a tree-to-tree vertex because it does not have any children. An *internal vertex* is any vertex of out-degree $> 0$. An arc is a *tree arc* if it ends at a tree vertex; otherwise, the arc is a *reticulation arc*. The *out-degree* and *in-degree* of any vertex *v* are denoted $d^{+}(v)$ and $d^{-}(v)$, respectively.

A network is *separated* if all its reticulate vertices have out-degree 1.

### Phylogenetic networks

A *phylogenetic network* on a set of *X* of distinctly labeled species is a rooted network $N=(V,A)$ such that $X=\{v \in V: d^{+}(v)=0, d^{-}(v)=1\}$ is a set of leaves. A *phylogenetic tree* is a phylogenetic network that has no reticulate vertices.

A *semi-binary* phylogenetic network is a separated network which has the properties that (i) each non-leaf tree vertex has out-degree $\ge 2$ and (ii) each reticulate vertex has in-degree 2.

A *binary* phylogenetic network is a separated phylogenetic network that has the property that each non-leaf tree vertex has out-degree 2 and each reticulate vertex has in-degree 2.

A phylogenetic network is *non-binary* if it is a non-separated network (i.e., there is at least one reticulate vertex with out-degree $> 1$).

A *tree-child network* is a phylogenetic network that has the property that each non-leaf vertex has a child that is a tree vertex.

A *normal* network is a tree-child network *N* with the additional property that if $$v_1,\cdots , v_k$$ is a directed path in *N* from $$v_1$$ to $$v_k$$ and $$ k > 2$$, then $$ (v_1, v_k)$$ is not an arc in *N* (i.e., there are no ‘short-cut’ arcs).

Let $N=(V,A)$ be a phylogenetic network. Define a relation $\mathrel {R}$ on the set $\overset{\circ }{V}$ of internal vertices of *N* by: $u\mathrel {R}v \Leftrightarrow u = v$, or *u* and *v* are linked only by reticulation arc(s) if we ignore the direction of arc(s).

The proof of the following result is straightforward and provided in the Appendix.

#### Lemma 1

If $N=(V,A)$ is a phylogenetic network, $\mathrel {R}$ is an equivalence relation on $\overset{\circ }{V}$.

We call the equivalence classes of $\mathrel {R}$ the *events* of *N* and write $\bar{u}$ for the equivalence class of a vertex *u*.

- Either $\bar{u} = \{u\}$, in which case $\bar{u}$ is called a *branching event*; or
- $\bar{u}$ has at least three elements, and $\bar{u}$ is called a *reticulation event*.

Given a phylogenetic network $N=(V,A)$, let

1 $$\begin{aligned} dN=\{\bar{v} :v\in \overset{\circ }{V}, v \ \text {is a reticulate vertex or a tree-to-tree vertex}\} \end{aligned}$$

be the set of equivalence classes of $\mathrel {R}$.

For two events $\bar{u}, \bar{v}$ of *N*, we say that $\bar{u}$ is descendant of $\bar{v}$ or $\bar{v}$ is ancestor of $\bar{u}$, denoted as $\bar{v}\preceq \bar{u}$, if there exist $v\in \bar{v}$ and $u\in \bar{u}$ such that $v\preceq u$ holds. We say that two events $\bar{v}$, $\bar{u}$ are $\preceq$-*comparable* if $\bar{v}\preceq \bar{u}$ or $\bar{u}\preceq \bar{v}$. Note that $\bar{u}\preceq \bar{u}$ trivially always holds for any event $\bar{u}$.

A phylogenetic network $N=(V,A)$ is said to have a *temporal labelling* if there is a function $t: V \rightarrow \mathbb {R}^{\ge 0}$ for which the following two properties hold:

- T1: if (*u*, *v*) is a reticulation arc, then $t(u) = t(v )$;
- T2: if (*u*, *v*) is a tree arc, then $t(u) < t(v )$.

If a phylogenetic network $N=(V,A)$ has a temporal labelling, then there is a function *r* (called a *ranking*) taking values from the set $\{0,1,2,\cdots , {e_{N}-1}\}$ where $e_N$ is the number of events of *N* with the root vertex assigned rank 0 (i.e., $r(\rho ) = 0$) and satisfying the following property: for each internal vertex *v*, if (*u*, *v*) is a tree arc, then $r(u) < r(v)$, and if *v* is a reticulate vertex, then *v* has the same rank as all internal vertices that are linked by reticulation arcs. This is the only case where equal ranking occurs. Since all vertices $u \in \bar{u}$ have the same rank, we define

$$ r(\bar{u}) := r(u) \quad \text {for any } u \in \bar{u}. $$

In the context of biology, rankings indicate a possible historical interpretation of evolution with the conditions that all ancestral species (along with the hybrid they formed) involved in a hybridization event must have been extant at the same time (T1) and ancestral speciation implies a passage of time (T2) Kong et al. (2022). In phylogenetics, leaves are generally regarded as present taxa/species, while internal vertices represent ancestral species. Therefore, rankings generally refer to internal vertices.

A network *N* is *rankable* if it has at least one ranking. Two rankings $r_1, r_2$ of *N* are *distinct* if there exists at least one vertex *v* such that $r_1(v)\ne r_2(v)$. Note that a rankable network may have multiple distinct rankings. Given a network *N*, let $\psi (N)$ denote the number of rankings of *N*.

**Fig. 1:**
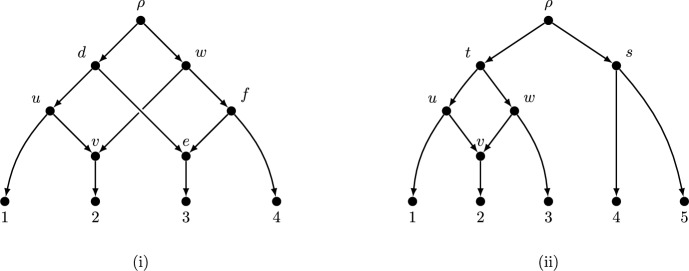
(i) A binary normal network which is not rankable. (ii) A binary rankable normal network with three distinct rankings.

Fig. 1(i) is an example of a binary normal network that has no ranking. To see why, suppose that a ranking function *r* exists. We would then have $r(u)=r(v)=r(w)=t_1$, and $r(d)=r(e)=r(f)=t_2$; in addition, we have $r(d)=t_2<r(u)=t_1$ (because (*d*, *u*) is a tree arc) and $r(w)=t_1<r(f)=t_2$ (because (*w*, *f*) is a tree arc). These last two inequalities $t_2<t_1<t_2$ provide a contradiction. On the other hand, Fig. 1(ii) is a binary rankable normal network and has three distinct rankings (i.e., we can let $r(s)<r(t)$ or $r(t)<r(s)<r(u)=r(v)=r(w)$, or $r(s)>r(u)=r(v)=r(w)$).

### Outline of results

It is known that every rankable binary tree-child network is normal; however, this does not extend to non-binary networks (we give a counterexample). Nevertheless, we show that separated tree-child networks that are rankable are normal.

In Section 3, we investigate a transformation to facilitate the enumeration of the rankings of any (binary or semi-binary) tree-child network (Proposition 3), thereby addressing a question posed at the end of Section 1.2 of Bienvenu et al. (2022).

Finally, we consider the expected number of rankings of a randomly-sampled binary tree-child network with *n* leaves and *k* reticulate vertices. We show this number is asymptotically of the form $\frac{1}{4^k} \cdot f(n)$ as *n* grows.

## Rankable separated tree-child networks are normal

Although every binary rankable tree-child network is normal (see e.g., Steel (2016) Prop. 10.12), non-binary rankable tree-child networks can fail to be normal, as we show shortly. Nevertheless, we also establish that every rankable separated tree-child network is normal.

**Fig. 2:**
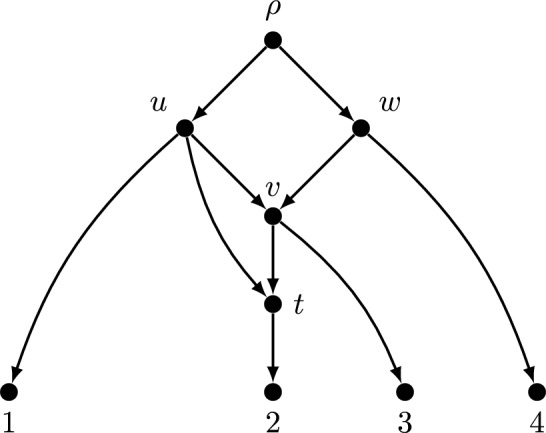
A non-binary tree-child network which has a temporal ordering but is not a normal network.

The network in Fig. 2 is a non-binary tree-child network (i.e., $d^{+}(v)=d^{-}(v)=2$) which has a temporal ordering but is not normal (since (*u*, *t*) is a short-cut arc). Note that *v* and *t* are both reticulate vertices. Although *t* is a child of *v*, the network is still a tree-child network because *v* has another leaf child (labelled as 3). We can temporally label all the vertices as follows: $r(\rho )=t_0=0, r(u)=r(w)=r(v)=r(t)=t_1$ and all the leaves have temporal label of $t_2$ such that $t_0<t_1<t_2$.

To prove the first result of the paper, we begin with the following lemma.

### Lemma 2

Given a rooted network *N* and a directed path $v_1,v_2,\cdots ,v_n$ of *N* and $n \ge 3$, if *N* has a temporal labelling, then $r(v_1)\le r(v_n)$ for any ranking function *r* and internal vertex $v_i$, $i\in \{1,2,\cdots ,n\}$.

### Proof

By definition of the ranking function, $r(v_i) \le r(v_{i+1})$ for every arc $(v_i, v_{i+1})$ on the directed path $v_1,v_2,\cdots ,v_n$. Therefore, $r(v_1) \le r(v_n)$.

### Proposition 1

If *N* is a separated tree-child network that has a temporal ordering, then *N* is normal.

### Proof

We provide a proof by contradiction. Suppose that *N* is a separated tree-child network that has a temporal ordering and *N* is not normal. *N* is temporal, so it has at least one ranking *r*. Then *N* has a directed path $v_1,v_2,\cdots ,v_k$ such that $k \ge 3$ and $(v_1,v_k)$ is an arc. Note that $v_k$ is a reticulate vertex such that $v_1$ and $v_{k-1}$ are parents of $v_k$; let $r(v_1)=r(v_{k-1})=r(v_k)=t_1$. Consider the vertex $v_{k-1}$. If $v_{k-1}$ is a reticulate vertex, then $v_{k-1}$ has exactly one child $v_k$, which is a reticulate vertex, and thus *N* is not a tree-child network, which is a contradiction. Therefore, $v_{k-1}$ is a tree vertex and $r(v_{k-2})<r(v_{k-1})=t_1$. In addition, $v_1,\cdots ,v_{k-1}$ is a directed path and, by Lemma 2, $r(v_1)=t_1\le r(v_{k-2})$, which is a contradiction.

## Counting the rankings of (separated) tree-child networks

Given a rooted tree $T=(V,A)$, a standard result in enumerative combinatorics (e.g. Donald Ervin Knuth (1997)) is the following:

2 $$\begin{aligned} \delta (T)=\frac{\vert V\vert !}{\prod _{v \in V}\lambda (v)}, \end{aligned}$$

where $\lambda (v)=\vert \{u\in V: v \preceq u\} \vert$ and $\preceq$ is the partial order defined at the start of Section 1.1.

Here we note that the number of events (as defined in Section 1.2) of *T* is just the number of internal vertices of *T*, and counting rankings of *T* is equivalent to counting topological orderings of the tree $T'=(V',A')$ obtained from *T* by deleting leaves and their incident arcs. Therefore,

$$\psi (T)=\delta (T')=\frac{\vert V'\vert !}{\prod _{v \in V'}\lambda (v)}.$$

However, for networks with reticulate vertices, the two concepts are different, and counting topological orderings is known to be #P hard for general networks Brightwell and Winkler (1991).

Consider a separated tree-child network $N=(V,A)$ with at least one reticulate vertex. We obtain an associated directed graph $\Psi (N)$ as follows. The vertex set of $\Psi (N)$ is *dN*, and for any two equivalence classes $\bar{u}$ and $\bar{v}$ of *dN*, $\bar{u}$ and $\bar{v}$ are joined by an arc $(\bar{u}, \bar{v})$ if there exist $t\in \bar{u}$ and $s\in \bar{v}$ such that (*t*, *s*) is a tree arc of *N* Baroni et al. (2006).

We denote the result of applying the above operations by writing

$$\begin{aligned} \Psi (N)= \tilde{N}. \end{aligned}$$

**Fig. 3:**
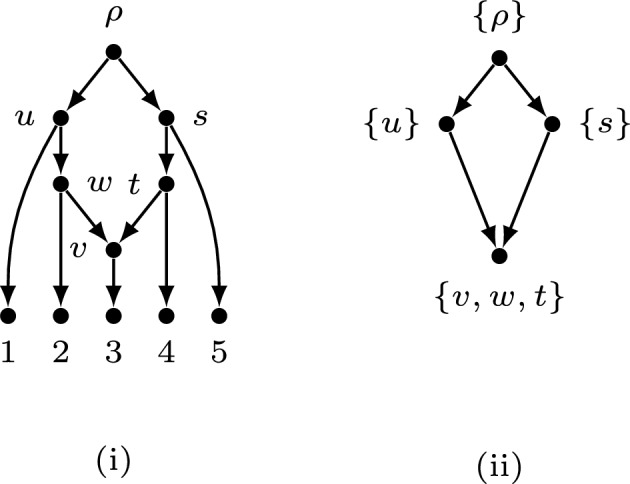
(i) A separated tree-child network *N* with five leaves and one reticulate vertex. (ii) The associated directed network $\Psi (N)$.

In the following, we will show that the number of rankings of *N* is equal to the number of topological orderings of $\Psi (N)$, and we illustrate this on a few examples. As seen in Fig. 3, (i) is a separated tree-child network *N* with five leaves and one reticulate vertex. The associated directed graph $\Psi (N)$ is shown in (ii) with vertex set of $\{\{\rho \},\{u\},\{s\},\{v,w,t\}\}$.

Note that *N* has two rankings (we can either let $r(u)<r(s)$ or $r(u)>r(s)$), and it is clear to see that $\Psi (N)$ has two topological orderings.

**Fig. 4:**
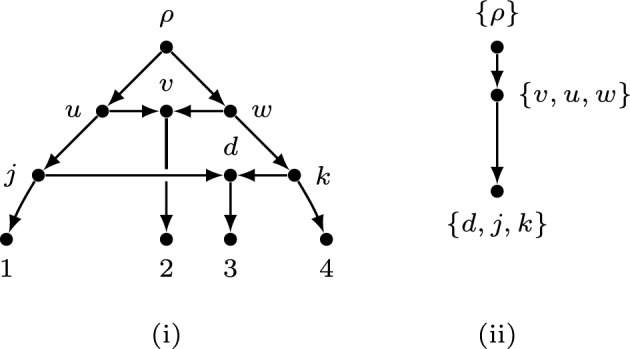
(i) A binary tree-child network *N* with four leaves and two reticulate vertices. (ii) The associated directed graph $\Psi (N)$.

Note that in some cases, $\Psi (N)$ might be a rooted tree. Fig. 4(i) is a binary tree-child network *N* with four leaves and two reticulate vertices. Fig. 4(ii) is the associated directed graph $\Psi (N)$ which is a rooted tree. By Eqn. (2) $\Psi (N)$ has exactly one topological ordering. Therefore, the network in Fig. 4 (i) has exactly one ranking as well.

### Proposition 2

Given a separated normal network $N=(V,A)$ with at least one reticulate vertex, if $\Psi (N)$ is a cyclic directed graph, then *N* does not have a ranking.

### Proof

If $\Psi (N)$ is a cyclic directed graph, then there exist two events $\bar{u}$ and $\bar{v}$ in *N* such that $\bar{u}$ is both a descendant and an ancestor of $\bar{v}$. In this case, *N* does not have a temporal labelling, and hence *N* does not have a ranking.

**Fig. 5:**
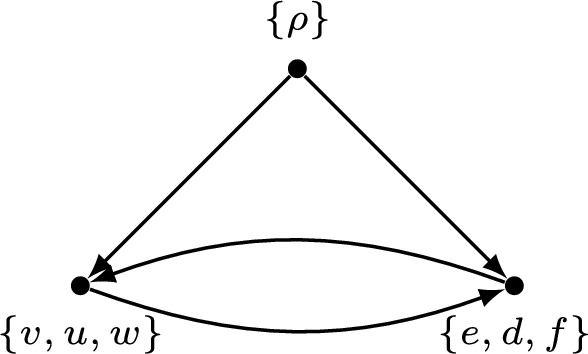
The associated directed graph $\Psi (N)$ of the network *N* (in Fig. 1 (i)) has a cycle.

As an example of Proposition 2, the separated normal network *N* in Fig. 1 (i) does not have a ranking. The associated directed graph in Fig. 5 has a cycle which means that there are two distinct events $\bar{v}$ and $\bar{e}$ such that $\bar{v} \prec \bar{e}$ and $\bar{e} \prec \bar{v}$.

### Proposition 3

Given a rankable separated tree-child network $N=(V,A)$, the number of possible rankings of *N* equals the number of topological orderings of $\Psi (N)=(\tilde{V}, \tilde{A})$. If $\Psi (N)$ is a rooted tree, we have:

$$\begin{aligned} \psi (N)=\delta (\Psi (N))=\frac{\vert \tilde{V}\vert !}{\prod _{v \in \tilde{V}}\lambda (v)}, \end{aligned}$$

where $\lambda (v)$ is the number of vertices in $\tilde{V}$ reachable from *v* by a directed path (including *v*).

### Proof

Let $\mathcal {K}(N)$ denote the set of rankings of *N*, and $\mathcal {T}(\Psi (N))$ the set of topological orderings of $\Psi (N)$.

Define a function

$$ f :\mathcal {K}(N) \rightarrow \mathcal {T}(\Psi (N)), \quad f(r) = \tau , $$

where $\tau$ is the ordering of $\Psi (N)$ satisfying

$$ r(\bar{u}) = \operatorname {ind}_\tau (\bar{u}) - 1 \quad \text {for each event } \bar{u} \text { of } N. $$

Then for each $r \in \mathcal {K}(N)$, there is a unique $\tau \in \mathcal {T}(\Psi (N))$ sorting the vertices according to their ranks in *N*. Therefore, *f* is well defined.

Next, we show that *f* is a bijection. Suppose $r_1, r_2 \in \mathcal {K}(N)$ are distinct but $f(r_1) = f(r_2) = \tau$. Then there exists an event $\bar{u}$ of *N* with $r_1(\bar{u}) \ne r_2(\bar{u})$. However, by definition of *f*, we have

$$ r_1(\bar{u}) = \operatorname {ind}_\tau (\bar{u}) - 1 = r_2(\bar{u}), $$

a contradiction. Therefore, *f* is injective.

For each $\tau \in \mathcal {T}(\Psi (N))$, define $r \in \mathcal {K}(N)$ by

$$ r(\bar{u}) = \operatorname {ind}_\tau (\bar{u}) - 1 \quad \text {for all events } \bar{u} \text { of } N. $$

Then $f(r) = \tau$, so *f* is surjective. Together with injectivity, this shows that *f* is a bijection. Hence, the number of rankings of *N* is the number of topological orderings of $\Psi (N)$. If $\Psi (N)$ is a rooted tree, the number of topological orderings of $\Psi (N)$, $\delta (\Psi (N))$, is given by Eqn. (2):

$$\begin{aligned} \delta (\Psi (N))=\frac{\vert \tilde{V}\vert !}{\prod _{v \in \tilde{V}}\lambda (v)}, \end{aligned}$$

which is the number of rankings of the separated tree-child network *N*.

**Remarks:** A phylogenetic network without any reticulate vertex is just a rooted tree. The number of rankings of a rooted binary tree $T=(V,A)$ with *n* leaves is

$$\begin{aligned} \frac{(n-1)!}{\prod _{v \in \tilde{V}}\lambda (v)}, \end{aligned}$$

where $\tilde{T}=(\tilde{V}, \tilde{A})$ is the rooted binary tree obtained by deleting all leaves of *T*, and $\lambda (v)$ is the number of vertices of $\tilde{T}$ descended from *v* (including *v*). The number of rankings of a rooted binary tree *T* is just the number of topological orderings of $\tilde{T}$, and the number of vertices of $\tilde{T}$ is $n-1$. In particular, certain rooted binary trees have exactly one ranking. More precisely a rooted binary tree has exactly one ranking if and only if it is a *caterpillar tree*, in which all the interior vertices form a directed path.

## The number of rankings of a random binary tree-child network

Let $X_{n,k}$ be the random variable that describes the number of rankings of a binary tree-child network on leaf set $[n]=\{1, \ldots , n\}$ with *k* reticulate vertices, chosen uniformly at random. Here *k* is fixed, and we let *n* grow.

The following result reveals that asymptotically (as *n* grows) the expected number of rankings asymptotically splits into a product of two functions; one involving just *k*, the other just *n*. Moreover, for any fixed *k*, it becomes increasingly certain that a random binary tree-child network will have at least one ranking as *n* grows.

### Proposition 4

For each fixed $k \ge 0$, as $n \rightarrow \infty$, the following hold:

(i) $$ \mathbb {E}[X_{n,k}] \sim \frac{1}{4^k} \cdot \frac{n!}{ \left( {\begin{array}{c}2n-2\\ n-1\end{array}}\right) }.$$
  In particular, $\lim _{n \rightarrow \infty } \frac{\mathbb {E}[X_{n,{k+1}}]}{\mathbb {E}[X_{n,k}]}= \frac{1}{4}$, for each $k \ge 1$.
(ii) $$\lim _{n \rightarrow \infty } \mathbb {P}(X_{n,k}\ge 1) =1.$$

### Proof

*Part (i)* If a tree-child network is chosen uniformly at random, then:

3 $$\begin{aligned} \mathbb {E}[X_{n,k}] = \frac{RTCN(n,k)}{TCN(n,k)}. \end{aligned}$$

where *RTCN*(*n*, *k*) denotes the number of ranked tree-child networks on the leaf set [*n*] with *k* reticulate vertices (i.e., the number of ordered pairs (*T*, *r*), where *T* is a tree-child network, and *r* is a ranking of the vertices of *T*) and *TCN*(*n*, *k*) denotes the number of tree-child networks on the leaf set [*n*] with *k* reticulate vertices.

From Bienvenu et al. (2022) (Theorem 1), we have:

4 $$\begin{aligned} RTCN(n,k)= \begin{bmatrix} n-1\\ n-1-k \end{bmatrix} \cdot \frac{n!(n-1)!}{2^{n-1}}\end{aligned}$$

where $$ \begin{bmatrix} n-1\\ n-1-k \end{bmatrix}$$ refers to the unsigned Stirling number of the first kind (i.e., the number of permutations on $n-1$ elements that have $n-1-k$ cycles). For *k* fixed, and as $n \rightarrow \infty$, we have the following result from Moser and Wyman (1958) (Eqn. 1.6):

5 $$\begin{aligned} \begin{bmatrix} n-1\\ n-1-k \end{bmatrix} \sim \frac{(n-1-k)^{2k}}{2^k k!} \end{aligned}$$

Note that the second term in Eqn. (4), namely, $\frac{n!(n-1)!}{2^{n-1}}$, is the number of ranked rooted binary trees on leaf set [*n*].

Moreover, for any fixed values of *k*, we have the following asymptotic equivalence as $n\rightarrow \infty$ from Fuchs et al. (2025):

6 $$\begin{aligned} TCN(n,k) \sim \frac{(2n^2)^k}{k!} r(n), \end{aligned}$$

where

7 $$\begin{aligned} r(n) = \frac{(2n-2)!}{(n-1)!2^{n-1}} \end{aligned}$$

is the number of rooted binary phylogenetic trees on leaf set [*n*].

Applying Eqns. (3) – (7), and noting that $(n-1-k)^{2k}/n^{2k} \rightarrow 1$ as $n\rightarrow \infty$ (for fixed *k*) gives the claimed result.

*Part (ii)* This follows from results in Fuchs et al. (2025), which show that the proportion of binary tree-child networks with *k* reticulate vertices and *n* leaves that have at least one ranking tends to 1 as $n \rightarrow \infty$.

## Concluding comments

In this paper, we have shown that every rankable separated tree-child networks is a normal network by establishing that for any rankable network *N* with a directed path $v_1, v_2, \cdots , v_n$ ($n\ge 3$), the temporal labelling of $v_1$ is at most that of $v_n$. Furthermore, given a separated tree-child network $N=(V,A)$, we can transform *N* to a simpler network $\Psi (N)$. We have defined a function from the set of rankings of *N* to the set of topological orderings of $\Psi (N)$ and shown that it is bijective. Consequently, counting the number of rankings of *N* is equivalent to counting the number of topological orderings of $\Psi (N)$, which is a standard problem in enumerative combinatorics Donald Ervin Knuth (1997) and efficiently solvable when $\Psi (N)$ is a tree. A possible question for future work could be to determine whether the problem of counting the number of rankings of separated tree-child network is $\#P$-complete.

Finally, we have investigated the expected number of rankings of a tree-child network with *n* leaves and *k* reticulate vertices selected uniformly at random, revealing a curious and simple asymptotic factorization into the product of a term involving just *k* and a term involving just *n*. Describing the asymptotic distribution of $X_{n,k}$ (suitably normalised) could be an interesting question for further work.

## Appendix: Proof of Lemma 1

For any vertex $v \in \overset{\circ }{V}$, we have $v\mathrel {R}v$, so $\mathrel {R}$ is reflexive. Moreover, for any $v, u \in \overset{\circ }{V}$, if $v\mathrel {R}u$, then either $u = v$, or *u* and *v* are linked only by reticulation arc(s). Therefore, we also have $u\mathrel {R}v$, and thus $\mathrel {R}$ is symmetric. Thus, it remains to establish transitivity of $\mathrel {R}$.

Suppose that for $v, u, t \in \overset{\circ }{V}$, we have $v\mathrel {R}u$ and $u\mathrel {R}t$. We claim that $v \mathrel {R}t$. This holds trivially if $u=v$, while if $u \ne v$, *u* and *v* are linked by reticulation arcs. Given that $u\mathrel {R}t$, if $u=t$, then $v \mathrel {R}t$; if *t* and *u* are linked by reticulation arcs, then *u* and *t* are linked by reticulation arcs too. Thus, we have $v\mathrel {R}t$.

In summary, for any $v, u, t \in \overset{\circ }{V}$, if $v\mathrel {R}u$ and $u\mathrel {R}t$, then $v \mathrel {R}t$, thus $\mathrel {R}$ is transitive, and so $\mathrel {R}$ is an equivalence relation on $\overset{\circ }{V}$.
